# A robust Parkinson’s disease detection model based on time-varying synaptic efficacy function in spiking neural network

**DOI:** 10.1186/s12883-024-04001-7

**Published:** 2024-12-30

**Authors:** Priya Das, Sarita Nanda, Ganapati Panda, Sujata Dash, Amel Ksibi, Shrooq Alsenan, Wided Bouchelligua, Saurav Mallik

**Affiliations:** 1 School of Electronics Engineering, Kalinga Institute of Industrial Technology, Bhubaneswar, India; 2 School of Electronics Engineering, C. V. Raman Global University, Bhubaneswar, India; 3 Information Technology Department, School of Engineering and Technology, Nagaland University, Kohima Campus, Meriema, India; 4 Information Systems Department, College of Computer and Information Sciences, Princess Nourah bint Abdulrahman University, P.O. Box 84428, Riyadh, 11671 Saudi Arabia; 5 Applied College, Imam Mohammad Ibn Saud Islamic University (IMSIU), Huraymila, Riyadh, 11432 Saudi Arabia; 6 Department of Environmental Health, Harvard T H Chan School of Public Health, Boston, MA 02115 USA; 7 Department of Pharmacology & Toxicology, University of Arizona, Tucson, MA 85721 USA

**Keywords:** Parkinson’s disease, Spiking neural network, Time-varying synaptic efficacy function, SEFRON, Spike timing dependent plasticity (STDP)

## Abstract

Parkinson’s disease (PD) is a neurodegenerative disease affecting millions of people around the world. Conventional PD detection algorithms are generally based on first and second-generation artificial neural network (ANN) models which consume high energy and have complex architecture. Considering these limitations, a time-varying synaptic efficacy function based leaky-integrate and fire neuron model, called SEFRON is used for the detection of PD. SEFRON explores the advantages of Spiking Neural Network (SNN) which is suitable for neuromorphic devices. To evaluate the performance of SEFRON, 2 publicly available standard datasets, namely (1) UCI: Oxford Parkinson’s Disease Detection Dataset and (2) UCI: Parkinson Dataset with replicated acoustic features are used. The performance is compared with other well-known neural network models: Multilayer Perceptron Neural Network (MLP-NN), Radial Basis Function Neural Network (RBF-NN), Recurrent Neural Network (RNN) and Long short-term memory (LSTM). The experimental results demonstrate that the SEFRON classifier achieves a maximum accuracy of 100% and an average accuracy of 99.49% on dataset 1. For dataset 2, it attains a peak accuracy of 94% and an average accuracy of 91.94%, outperforming the other classifiers in both cases. From the performance, it is proved that the presented model can help to develop a robust automated PD detection device that can assist the physicians to diagnose the disease at its early stage.

## Introduction

Parkinson’s disease is a neurological disorder that causes unexpected or uncontrollable movements, such as tremors, stiffness, and balance and coordination problems. Parkinson’s disease is the second most common neurodegenerative disorder after Alzheimer’s disease with more than 10 million people worldwide having PD [1]. Since 1990, the death rate from Parkinson’s disease per 100,000 people in India has increased by 87.9%, with an annual average of 3.8% [2]. Dopamine plays an important role in controlling body movements. When neurons die or are damaged, they produce less dopamine, causing movement disorders [3]. Parkinson’s disease is known to cause a loss of automatic movements. This includes a reduced ability to perform unconscious actions like blinking, smiling, or swinging the arms while walking. This symptom arises due to the overall decline in motor function and coordination that characterizes the disease, impacting both voluntary and involuntary movements; also, changes in speech and writing may occur. As there is no cure for PD, early detection can help control the symptoms. The statistics show that the number of PD patients is increasing at an alarming rate and to differentiate PD patients from healthy individuals, thorough examinations, including EEG recordings, video observations, and speech assessments, may be required, particularly in less severe or ambiguous cases. In more advanced PD cases, clinical symptoms may be more evident, requiring fewer diagnostic procedures. Therefore, automation can be a relief for doctors from such tremendous pressure. Hence, in this paper, we proposed an accurate, robust, and fast model for the automatic detection of PD.

Due to the increasing number of PD patients, different researchers have applied various artificial neural network (ANN) techniques for the detection of PD. They have used a multitude of data modalities including voice recordings [4], movement [5], magnetic resonance imaging (MRI) [6], handwriting [7], single-photon emission computed tomography (SPECT) [8]. However, among all the bio-markers, voice data plays a significant role in the detection of PD as change in voice is one of the early symptoms of PD. Studies have shown that vocal features such as reduced volume, monotone speech, and imprecise articulation can be detected long before the motor symptoms like tremors and gait disturbances become apparent [9]. Voice data collection is non-invasive, requiring only a microphone and recording environment, making it significantly less expensive and more accessible than MR imaging, which requires costly and sophisticated equipment [10]. Advanced machine learning models have shown high accuracy in classifying PD using voice data. Algorithms such as Support Vector Machines (SVM), Random Forests, and Deep Neural Networks have been effectively applied to vocal features, achieving impressive results [11]. While MR imaging and gait features are also valuable, they often require more specialized equipment and conditions for accurate measurement. Moreover, MR imaging might not always be accessible for regular monitoring [12]. Voice data has shown consistent results across different studies and populations. However, it is essential for studies to analyze similar PD groups (in terms of sex percentages, ages, disease severity, medication, etc.) to ensure uniformity and reliability of results [13]. Sakar et al. [14] applied Support Vector Machine (SVM) and k-nearest neighbour (KNN) techniques on the voice recordings collected from 40 subjects including PD patients and healthy subjects. They achieved an average accuracy of 55% using the cross-validation technique. The accuracy was improved using different feature extraction, feature selection and classification methods [15–17]. In the paper [18], the ensemble model with SVM and Random Forest (RF) classifier is used. The weights of the model are optimized using Particle Swarm Optimization (PSO) Techniques. Although, the average and maximum accuracy of the model are 93.7% and 100% consecutively, the computational cost is high. Models based on the Extreme Learning Machine classifier [19] have a comparatively fast response time. Several Deep Neural Network (DNN) [20–22] based models are also explored for the diagnosis of PD patients. Convolutional Neural network AlexNet obtained 88.9% accuracy [21] from Magnetic Resonance (MR) images. In the paper [22], a long short-term memory (LSTM) network is applied on the gait pattern and obtained an average accuracy of 98.6%. Quan et al. [23] used Bidirectional LSTM combining the dynamic articulation transition features for PD detection and achieved 75.56% accuracy. This study focused on exploring time-series characteristics of continuous speech signals. Recurrent Neural Network with LSTM [24] has also been explored to analyze voice features. The authors obtain 95.8% accuracy on the parkinson’s telemonitoring voice dataset from the UCI public repository of datasets.

Although ANN or DNN models have produced good results in classification and pattern recognition, the rapid growth of neuromorphic engineering and increasing demand of high-performance computational hardware require more advanced, faster, and energy-conservative neural networks. Hence, Spiking Neural Network (SNN) is presented as the third generation of neural network architectures [25]. Unlike the previous ANN and DNN models, in SNN, the information propagates between different neurons in the form of spikes which is more like the human brain. DNNs transmit information between neurons using real numbers, whereas SNNs rely on 1-bit spikes for communication. SNN requires a smaller number of neurons to approximate a function in comparison to previous ANN and DNN models resulting higher computational power [26]. SNN has an intriguing property that output spike trains can be sparse in time and only fires when the post-synaptic potential reaches a certain threshold. Few spikes with high information content consume less energy making the SNN model energy-efficient [27]. Researchers have developed different supervised learning algorithms in the field of SNN such as SpikeProp [28], tempotron [29], synaptic weight association training (SWAT) [30] and others [31, 32]. The algorithms are modelled on biologically relevant mathematical neural models which include Hodgkin-Huxley (HH) model [33], the Leaky-Integrate-and-Fire (LIF) models [34], Izhikerich’s (Iz) model [35], and Spike Response Model (SRM) [36]. The most popular model is the LIF model due to its simple but efficient structure. The inputs fed to the SNN model are spikes in nature which can compress the data size in comparison to real-world analog data. There are various analog-to-spike encoding schemes including population encoding [28], rate encoding [32] and temporal encoding [37]. In SNN, the input neurons having pre-synaptic potentials or input spikes relate to output neurons through synapse model. The strength (or synaptic weights) between input and output neurons determines the probability of firing of output spikes, also called post-synaptic potentials. There are two types of synaptic plasticity: Long-term-plasticity (constant weight) [38] and short-term-plasticity (dynamic weights) [39]. Short-term-plasticity has more computational power than long-term-plasticity.

 SNN is an emerging research area in the medical domain offering promising results [40–43]. Virgilio et al. [40] used SNN with Izhikevich model and trained the model using the Particle Swarm Optimization (PSO) algorithm to classify motor imagery tasks from EEG signals. They obtained an average accuracy of 88.75% accuracy. Also, Rajagopal et al. [43] applied deep convolutional SNN for the detection of lung disease. Although PD detection is a well-studied research topic as an application of first and second-generation ANNs, little work has been done by applying SNN algorithms [44–46]. The authors [44] have applied evolutionary SNN to classify PD and healthy persons using the SRM Model with the grammatical evolution (GE) algorithm and achieved 85.96% accuracy. In this paper, an SNN based supervised learning algorithm with time-varying synaptic efficacy function and LIF neuron, called SEFRON [47] is presented for PD detection. In SEFRON, the weights are represented by adding different amplitude-modulated Gaussian distribution functions within a specific time window; thus, giving dynamic plasticity to the model. SEFRON has shown promising results for other diseases producing high accuracy and less computational complexity. In this article, UCI: Oxford Parkinson’s Disease Detection Dataset and UCI: Parkinson Dataset with replicated acoustic features (described in Sect. 3) are used to evaluate the performance of the SEFRON model and the outcomes are compared with well-known classifiers: MLP [48], RBF [49], RNN [50] and LSTM [51]. The objectives of the paper are:

- To implement an energy-efficient Spiking Neural Network-based SEFRON model for the detection of Parkinson’s disease.
- To obtain high accuracy for balanced and unbalanced PD dataset.
- To compare the performance of the proposed SEFRON model with other SNN models as well as popular neural network (viz. MLP, RBF, RNN and LSTM) models for benchmark dataset.

The rest of the paper is organized as follows: Sect. 2 elaborates on the proposed method, Sect. 3 describes the datasets used and discusses the experimental results, and finally, Sect. 4 presents the conclusion and future work of the paper.

## Methodology for the detection of Parkinson’s disease

### Dataset description

In this paper, two publicly available standard UCI: Oxford Parkinson’s Disease Detection Dataset and UCI: Parkinson Dataset with replicated acoustic features are used to evaluate the performance of the suggested methods.

**Fig. 1:**
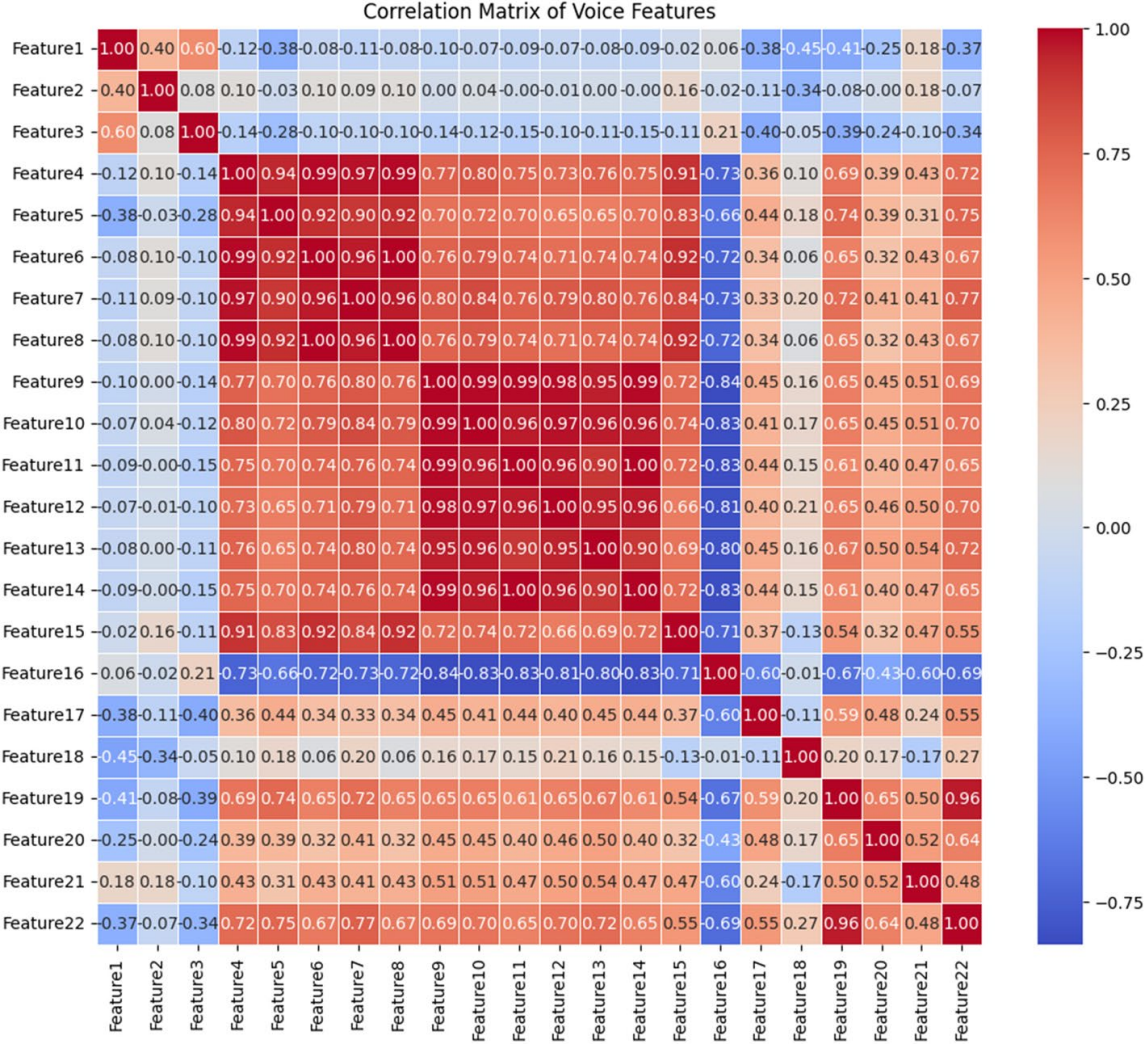
Correlation Matrix of the features present in dataset#1
B *Dataset 2*: This dataset [53] includes acoustic features extracted from sustained /a/ phonation voice recordings of 80 subjects, comprising 40 individuals with Parkinson’s Disease (PD). Among these subjects, 32 are female and 48 are male. Each subject has three replications, resulting in 240 instances in total, with 44 features per instance. The features include pitch and amplitude perturbation measures, harmonic-to-noise ratios, Mel frequency cepstral coefficients, and other specialized metrics like recurrence period density entropy and pitch period entropy.

A *Dataset 1:* This dataset [52] consists of speech measurements collected from 31 persons, 23 people are suffering from PD. There are 195 instances with 22 features and the last column signifies whether the person is PD patient or healthy (0 implies healthy and 1 implies PD). The details of the features are elaborated in Table 1. Fig. 1 shows the correlation matrix of features from dataset 1. From the correlation matrix, it is visible that feature 4 and 5, feature 9 and 10, and feature 13 and 14 have extremely high positive correlations with each other. This suggests that these features are likely providing similar information and might be redundant.

**Table 1:** Description of dataset #1

	Feature Name		PD			Healthy		
			Min Value	Max Value	Median	Min Value	Max Value	Median
1	MDVP: Fo (Hz)	Average vocal fundamental frequency	88.33	223.36	145.174	110.74	260.11	198.996
2	MDVP: Fhi (Hz)	Maximum vocal fundamental frequency	102.15	588.52	163.335	113.60	592.03	231.1615
3	MDVP: Flo (Hz)	Minimum vocal fundamental frequency	65.48	199.02	99.77	74.29	239.17	113.9385
4	MDVP: Jitter(%)	MDVP jitter in percentage	0.00168	0.03316	0.00544	0.00178	0.0136	0.00336
5	MDVP: Jitter (Abs)	MDVP absolute jitter in ms	0.00001	0.00026	0.00004	0.000007	0.00008	0.000025
6	MDVP: RAP	MDVP relative amplitude perturbation	0.00068	0.02144	0.00284	0.00092	0.00624	0.00163
7	MDVP: PPQ	MDVP five-point period perturbation quotient	0.00092	0.01958	0.00314	0.00106	0.00564	0.001775
8	Jitter: DDP	Average absolute difference of differences between jitter cycles	0.00204	0.06433	0.00853	0.00276	0.01873	0.00488
9	MDVP: Shimmer	MDVP local shimmer	0.01022	0.11908	0.02838	0.00954	0.04087	0.01671
10	MDVP: Shimmer (dB)	MDVP local shimmer in dB	0.09	1.302	0.263	0.085	0.405	0.154
11	Shimmer: APQ3	Three-point amplitude perturbation quotient	0.00455	0.05647	0.01484	0.00468	0.02336	0.00878
12	Shimmer: APQ5	Five-point amplitude perturbation quotient	0.0057	0.0794	0.0165	0.00606	0.02498	0.01023
13	MDVP: APQ	MDVP 11-point amplitude perturbation quotient	0.00811	0.13778	0.02157	0.00719	0.02745	0.01302
14	Shimmer: DDA	Average absolute differences between the amplitudes of consecutive periods	0.01364	0.16942	0.04451	0.01403	0.07008	0.02633
15	NHR	Noise-to-harmonics ratio	0.00231	0.31482	0.01658	0.00065	0.10715	0.00483
16	HNR	Harmonics-to-noise ratio	8.441	29.928	21.414	17.883	33.047	24.997
17	RPDE	Recurrence period density entropy measure	0.26365	0.68515	0.53053	0.25657	0.66384	0.43537
18	DFA	Signal fractal scaling exponent of detrended fluctuation analysis	0.57428	0.82529	0.72665	0.62671	0.78571	0.68253
19	spread1	Two nonlinear measures of fundamental	−7.12092	−2.43403	−5.44004	−6.75926	0.64278	−6.82645
20	spread2	Frequency variation	0.06341	0.45049	0.24088	0.00627	0.29195	0.16736
21	D2	Correlation dimension	1.76596	3.67116	2.43959	1.42329	2.88245	2.12951
22	PPE	Pitch period entropy	0.09319	0.52737	0.22272	0.04454	0.25240	0.11512

i) Pitch local perturbation measures: relative jitter (Jitter_rel), absolute jitter (Jitter_abs), relative average perturbation (Jitter_RAP), and pitch perturbation quotient (Jitter_PPQ).
ii) Amplitude perturbation measures: local shimmer (Shim_loc), shimmer in dB (Shim_dB), 3-point amplitude perturbation quotient (Shim_APQ3), 5-point amplitude perturbation quotient (Shim_APQ5), and 11-point amplitude perturbation quotient (Shim_APQ11).
iii) Harmonic-to-noise ratio measures: harmonic-to-noise ratio in the frequency band 0–500 Hz (HNR05), in 0–1500 Hz (HNR15), in 0–2500 Hz (HNR25), in 0–3500 Hz (HNR35), and in 0–3800 Hz (HNR38).
iv) Mel frequency cepstral coefficient-based spectral measures of order 0 to 12 (MFCC0, MFCC1,…, MFCC12) and their derivatives (Delta0, Delta1,…, Delta12).
v) Recurrence period density entropy (RPDE), vi) Detrended fluctuation analysis (DFA), vii) Pitch period entropy (PPE), and viii) Glottal-to-noise excitation ratio (GNE).

**Fig. 2:**
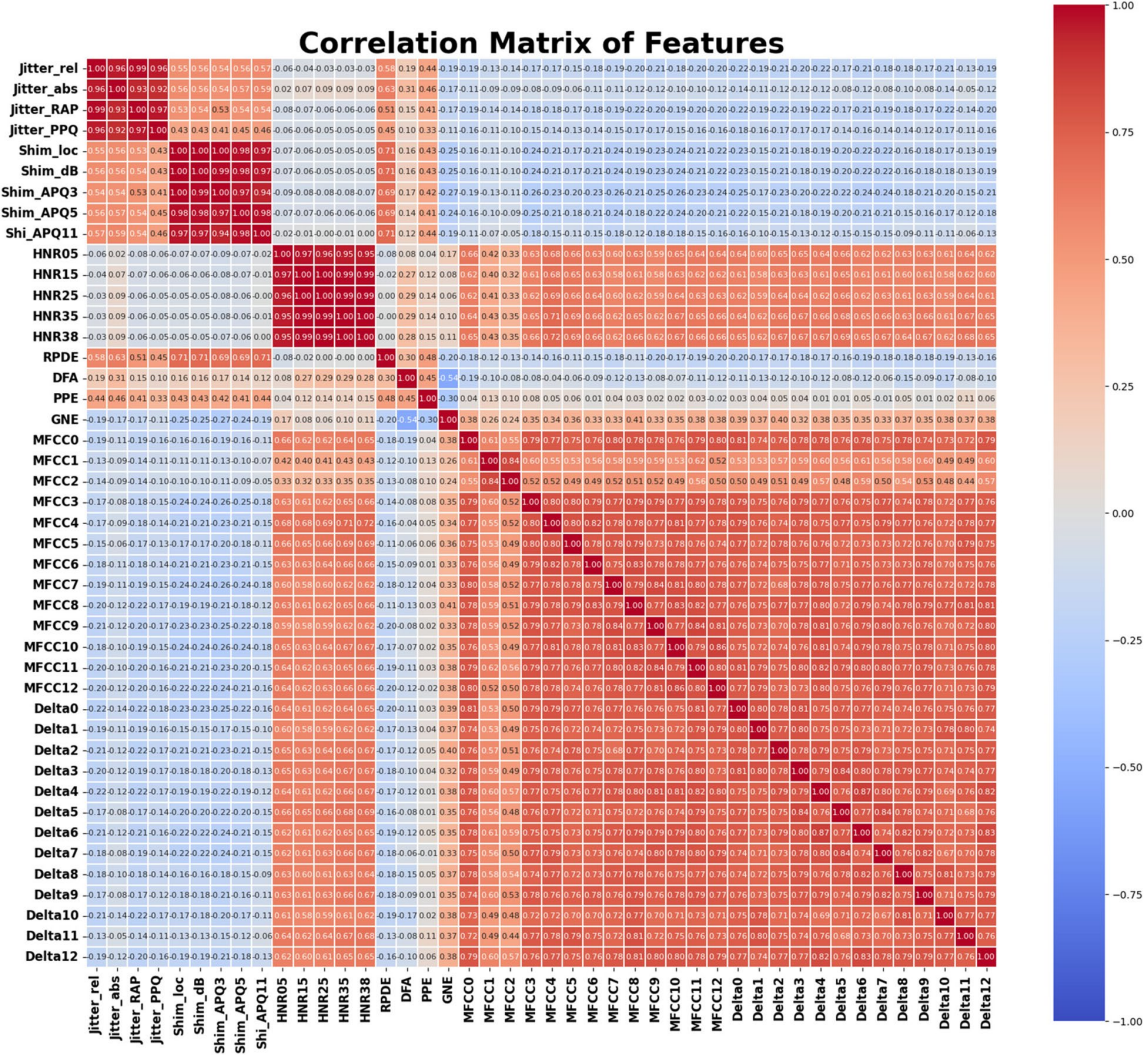
Correlation Matrix of the features present in dataset#2

Table 2 describes the range of each feature for both PD and healthy subjects. The correlation matrix of dataset 2 is shown in Fig. 2. Many features within certain groups show strong positive correlations, indicating they might be measuring similar characteristics or related aspects of the data. The MFCC features and Delta features (Delta1 through Delta12) exhibit high correlations with each other (values mostly in the range of 0.7–0.9). This suggests that these features are closely related and may provide redundant information. HNR05, HNR15, HNR25, HNR35, and HNR38 are also highly correlated with each other. These high correlations indicate that these features may be capturing similar characteristics of voice quality, specifically related to harmonics and noise levels. Jitter (e.g., Jitter_rel, Jitter_abs, etc.) and Shimmer features (e.g., Shim_loc, Shim_dB) generally show low to moderate correlations (values close to 0) with the MFCC, Delta, and HNR features. This suggests that the Jitter and Shimmer features may capture unique information about the voice data that is not covered by other feature groups. Some Jitter features have negative correlations with features like GNE (Glottal-to-Noise Excitation), though these correlations are generally weak or moderate.

**Table 2:** Description of dataset #2

	Feature Name	PD			Healthy		
		Min value	Max value	Median	Min value	Max value	Median
1	Jitter_rel	0.15406	6.8382	0.459265	0.14801	1.66	0.3209
2	Jitter_abs	0.00000707	0.00054986	0.0000353	0.00000816	0.0001548	0.0000169
3	Jitter_RAP	0.00094451	0.043843	0.0024889	0.0006783	0.010179	0.0018967
4	Jitter_PPQ	0.001193	0.065199	0.0027538	0.0010358	0.0093634	0.0020547
5	Shim_loc	0.013511	0.1926	0.030173	0.007444	0.075575	0.0373045
6	Shim_dB	0.11953	1.7476	0.261085	0.064989	0.67396	0.32909
7	Shim_APQ3	0.006986	0.11324	0.017387	0.0033436	0.043532	0.0210605
8	Shim_APQ5	0.0081477	0.12076	0.018711	0.004103	0.047809	0.0239305
9	Shi_APQ11	0.010865	0.14244	0.021451	0.006459	0.05853	0.0260115
10	HNR05	22.22472	85.82675	54.648028	44.04468	101.20633	66.771324
11	HNR15	26.27403	95.82331	61.115223	46.37354	109.65112	66.871476
12	HNR25	33.15610	105.63068	73.108972	55.82089	120.71283	76.758923
13	HNR35	36.49402	110.63571	79.768243	60.96113	128.28933	82.172395
14	HNR38	36.90821	111.48172	79.887769	61.96711	129.98524	82.484571
15	RPDE	0.16276	0.53595	1.347184	0.18697	0.46493	0.277062
16	DFA	0.41136	0.76973	1.347184	0.46278	0.78438	0.531063
17	PPE	0.00413	0.83583	1.347184	0.00453	0.90840	0.007481
18	GNE	0.85401	0.98616	1.347184	0.84731	0.98729	0.934484
19	MFCC0	0.77015	1.94910	1.347184	0.85355	1.82285	1.474352
20	MFCC1	0.72552	1.66621	1.357392	0.74028	1.83565	1.465031
21	MFCC2	0.58091	1.92843	1.276835	0.56947	1.86498	1.367316
22	MFCC3	0.72761	1.76753	1.308839	1.01980	1.85708	1.323482
23	MFCC4	0.77126	1.77313	1.316501	1.12197	1.84085	1.248497
24	MFCC5	0.61154	1.70349	1.272507	1.10621	1.97615	1.335238
25	MFCC6	0.82909	1.86082	1.354877	0.99518	2.00078	1.265518
26	MFCC7	0.85015	1.92178	1.328514	0.65356	2.01673	1.350374
27	MFCC8	0.83954	1.75379	1.27815	0.97761	1.91843	1.43609
28	MFCC9	0.82363	1.80147	1.311421	1.11741	2.03958	1.396401
29	MFCC10	0.81361	1.71300	1.310119	1.09603	2.07129	1.354033
30	MFCC11	0.82316	1.80121	1.364874	1.07187	1.98356	1.481258
31	MFCC12	0.84436	1.74245	1.338597	1.11141	2.02998	1.396623
32	Delta0	0.62084	1.81403	1.335865	0.85903	2.02806	1.480762
33	Delta1	0.64741	1.85068	1.355913	1.06983	2.02129	1.469876
34	Delta2	0.62811	1.74830	1.345888	0.64049	1.97986	1.505809
35	Delta3	0.76646	1.81060	1.366277	1.04844	1.86059	1.423438
36	Delta4	0.84013	1.83910	1.301322	1.10800	2.03824	1.535167
37	Delta5	0.74169	1.73507	1.378934	1.14310	1.78598	1.466678
38	Delta6	0.75969	1.87679	1.38297	1.09120	1.98809	1.476818
39	Delta7	0.76465	1.75499	1.347864	1.07393	1.87280	1.37731
40	Delta8	0.76280	1.83052	1.376092	1.07753	1.92013	1.369121
41	Delta9	0.81194	1.75996	1.234949	1.11208	1.94333	1.429185
42	Delta10	0.77701	1.92014	1.360012	1.09022	1.94968	1.353333
43	Delta11	0.64313	1.78491	1.358487	1.13137	1.91839	1.501565
44	Delta12	0.74841	1.85765	1.38134	1.13883	1.93010	1.397031

**Fig. 3:**
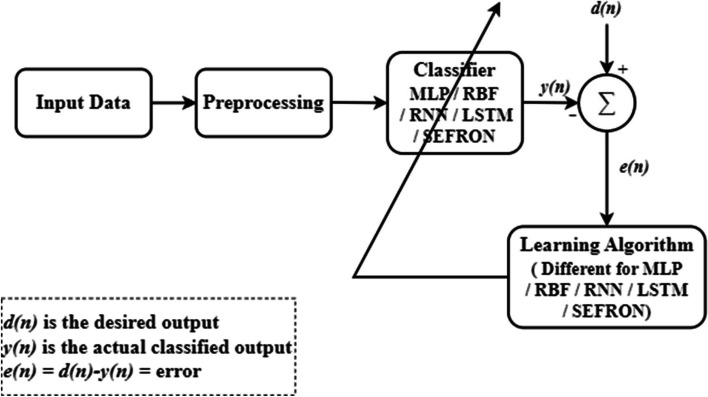
General block diagram for the detection of PD using neural network models

Figure 3 shows the proposed model for the detection of Parkinson’s disease. The model has three important stages: data collection, preprocessing and classification. The first PD dataset [52] discussed in Sect. 3 contains 22 features, while the second dataset [53] includes 44 features. The input data $\:{X}_{c}$ has $\:M\times\:N$ dimension where $\:M$ is the number of samples and $\:N$ signifies the number of features. The $\:{a}^{th}$ sample of the $\:{b}^{th}$ feature is normalized using Eq. 1.

1 $$\:X\left(a,b\right)=\frac{{X}_{c}(a,b)}{max\left({X}_{c}\left(:,b\right)\right)}$$

The normalized data $\:X$ is fed to the classifiers. In this paper, the performance of five classifiers: MLP, RBF, RNN, LSTM and SEFRON are compared. The parameters of the classifiers are adjusted based on the difference (e(n)) between the actual classified output (y(n)) and the desired output (d(n)), following their respective learning algorithms. Here, we outline the operational principle of the proposed classifier.

### Proposed SEFRON model

**Fig. 4:**
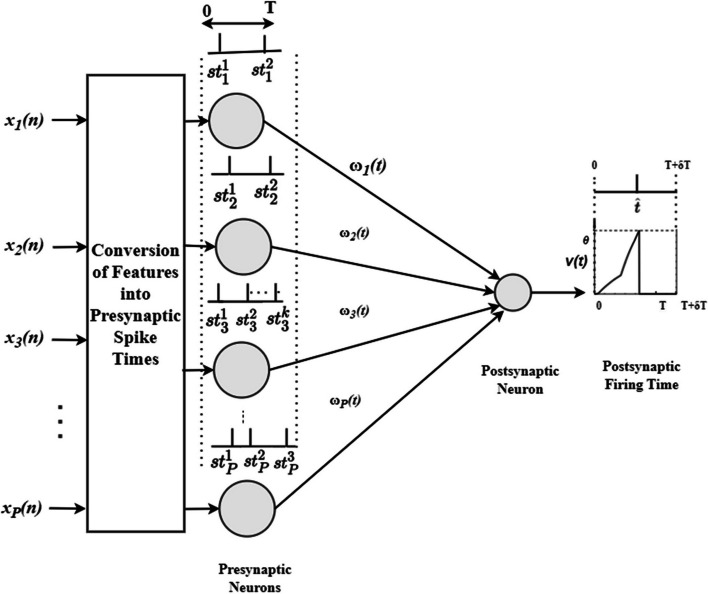
Architecture of SEFRON model with P number of presynaptic neuron and a single postsynaptic neuron

Figure 4 shows the SEFRON architecture with a single neuron showing both inhibitory and excitatory nature. Here,$\:\:P$ number of presynaptic neurons and a single output neuron are used. The presynaptic spikes can occur within $\:T\:ms$ time interval and $\:\left[T+{\updelta\:}\text{T}\right]ms$ time window is considered to capture the postsynaptic spike. Extended $\:{\updelta\:}\text{T}\:ms$ is considered for the late postsynaptic spikes. At first, the normalized input data (using Eq. (1)) is converted into presynaptic spike times using a well-studied population encoding scheme. The input features are fed to multiple receptive field neurons and each neuron generates one spike. The total number of spikes are determined by the number of receptive field neurons ($\:P$). The firing strength $\:\left({\psi\:}_{m}^{l}\right)$ of each receptive field neuron $l\;\left(l\;\epsilon\left[1,Q\right]\right)$ for the $\:{m}^{th}$ input is calculated using Eq. (2).

2 $$\:{\psi\:}_{m}^{l}={e}^{-\frac{{({x}_{m}-{\mu\:}_{l})}^{2}}{2{{\sigma\:}_{l}}^{2}}}$$

where $\:{\mu\:}_{l}$ and $\:{\sigma\:}_{l}$ are center and standard deviation of the $\:{l}^{th}$ receptive field neuron. $\:{\mu\:}_{l}$ and $\:{\sigma\:}_{l}$ are calculated as

3 $$\:{\mu\:}_{l}=\frac{(2 L-3)}{2(Q-2)}$$

4 $$\:{\sigma\:}_{l}=\frac{1}{\beta\:(Q-2)}$$

5 $$\:{t}_{j\_l}^{1}=T\times\:[1-{\psi\:}_{m}^{l}]$$

where $\:\beta\:$ is the overlap constant.

The set of presynaptic spike times $\:{\mathcal{F}}_{j}$ is defined as,

6 $$F_j=\left({st}_j^k,1\leq k\leq N_j\right),\;j\in\left\{1,\;P\right\}$$

where $\:{N}_{j}$ signifies the total number of spikes fired by the $\:{j}^{th}$ neuron. Each presynaptic neuron is connected to the postsynaptic neuron through time varying synaptic efficacy function or weights. The weights between $\:{j}^{th}$ presynaptic neuron and the postsynaptic neuron is denoted as $\:{\omega\:}_{j}\left(t\right)$ and the spike response function of a LIF neuron is represented by $\:\phi\:\left(t\right)$ as shown in Eq. 7.

7 $$\:\phi\:\left(t\right)=\frac{t}{\tau\:}{e}^{\left(1-\frac{t}{\tau\:\:}\:\right)}$$

where $\:\tau\:$ is the time constant. The postsynaptic potential $\:v\left(t\right)$ of SEFRON is defined as

8 $$\:v\left(t\right)=\sum\:_{j=1}^{P}\sum\:_{k=1}^{{N}_{j}}{\omega\:}_{j}\left({st}_{j}^{k}\right).\phi\:(t-{st}_{j}^{k})$$

Postsynaptic neuron fires when the postsynaptic potential $\:v\left(t\right)$ reaches to the threshold, $\:\theta\:$. If the firing time is $\:{t}_{f}$, $\:\theta\:$ is defined as

9 $$\:\theta\:=v\left({t}_{f}\right)=\sum\:_{j=1}^{P}\sum\:_{k=1}^{{N}_{j}}{\omega\:}_{j}\left({st}_{j}^{k}\right).\phi\:({t}_{f}-{st}_{j}^{k})$$

To update the synaptic efficacy functions, normalized form of STDP based learning rule is used. The rule defines the change in weights due to delay $\:x$ as

10 $$\:\delta\:\omega\:\left(x\right)=\left\{\begin{array}{c}+{V}_{+\:}{e}^{-\frac{x}{{\tau\:}_{+}}}\:if\:x\ge\:0\\\:-{V}_{-\:}{e}^{\frac{x}{{\tau\:}_{-}}}\:if\:x<0\end{array}\right.$$

$\:{V}_{+\:}$and $\:{V}_{-\:}$denote the highest change in weight due to long term potentiation and depression respectively. $\:{\tau\:}_{+}$ and $\:{\tau\:}_{-}$ represent the long-term potentiation and depression plasticity window respectively. As SEFRON considers only one postsynaptic spike, no presynaptic firing will be considered after first presynaptic firing. Hence, $\:{V}_{-\:}$is equal to zero here. The fractional contribution $\:{\chi\:}_{j}^{k}\left({t}_{f}\right)$ due to presynaptic spike at $\:{st}_{j}^{k}$ and postsynaptic spike $\:{t}_{f}$ is calculated as,

11 $$\:{\chi\:}_{j}^{k}\left({t}_{f}\right)=\frac{\delta\:\omega\:({t}_{f}-{st}_{j}^{k})}{\sum\:_{j=1}^{P}\sum\:_{k=1}^{{N}_{j}}\delta\:\omega\:({t}_{f}-{st}_{j}^{k})}$$

The fractional contribution points out the significance of a particular presynaptic spike. Higher is the value, more contribution the spike has to generate the postsynaptic spike. By replacing $\:{\omega\:}_{j}\left({st}_{j}^{k}\right)$ with $\:{\chi\:}_{j}^{k}\left({t}_{f}\right)$ in Eq. (8), postsynaptic potential is defined as,

12 $$\:{V}_{PSP}\left({t}_{f}\right)=\sum\:_{j=1}^{P}\sum\:_{k=1}^{{N}_{j}}{\chi\:}_{j}^{k}\left({t}_{f}\right).\phi\:({t}_{f}-{st}_{j}^{k})$$

As the value of postsynaptic potential varies with different input spikes, the ratio of threshold to postsynaptic potential which is referred as the overall strength is considered here. The error function $\epsilon$ is the difference between overall strength at desired $\:\left({t}_{fd}\right)$ and actual $\:{(t}_{fa})$ postsynaptic firing time (as shown in Eq. 13).

13 $$\epsilon={\lambda\:}_{{t}_{fd}}-{\lambda\:}_{{t}_{fa}}=\frac{\theta\:}{{V}_{PSP}\left({t}_{fd}\right)}-\frac{\theta\:}{{V}_{PSP}\left({t}_{fa}\right)}$$

Therefore, the change in synaptic efficacy function is determined by Eqs. (14, 15).

14 $$\:\varDelta\:{\omega\:}_{j}\left({st}_{j}^{k}\right)=\mu\:.{\chi\:}_{j}^{k}\left({t}_{fd}\right).\epsilon$$

15 $$\:\varDelta\:{\omega\:}_{j}\left({st}_{j}^{k}\right)=\mu\:.({\lambda\:}_{{t}_{fd}}.{\chi\:}_{j}^{k}({t}_{fd})-{\lambda\:}_{{t}_{fa}}.{\chi\:}_{j}^{k}({t}_{fd}\left)\right)$$

where $\:\mu\:$ is the learning rate. Now, $\:\varDelta\:{\omega\:}_{j}\left({st}_{j}^{k}\right)$ is modulated using Gaussian distribution function to incorporate the time varying nature of synaptic efficacy function.

16 $$\:{f}_{j}^{k}\left(t\right)=\varDelta\:{\omega\:}_{j}\left({st}_{j}^{k}\right).{e}^{-\frac{{(t-{st}_{j}^{k})}^{2}}{2{\sigma\:}^{2}}}$$

Here, σ is the efficacy update range. Finally, the updated weight for the $\:{j}^{th}$ neuron is determined by adding the contribution of time varying synaptic efficacy changes due to all presynaptic spikes of the particular neuron. The new synaptic efficacy function is given as,

17 $$\:{\omega\:}_{j\:new}\left(t\right)={\omega\:}_{j\:old}\left(t\right)\:+\sum\:_{k=1}^{{N}_{j}}{f}_{j}^{k}\left(t\right)$$

This model is used for two-class classification problem by assigning two appropriate values to the postsynaptic firing times. Assume, $\:{t}_{d1}$ and $\:{t}_{d2}$ are two different class labels, $\:{t}_{b}$ is the boundary between two classes and $\:y\left(n\right)$ is the calculated output for the $\:{n}^{th}$ input sample.

18 $$\:y\left(n\right)=\left\{\begin{array}{c}{t}_{d1},\:\:{t}_{a}<{t}_{b}\\\:{t}_{d2},\:\:{t}_{a}\ge\:{t}_{b}\end{array}\right.$$

19 $$\:y\left(n\right)=\left\{\begin{array}{c}class-1,\:\:{t}_{a}<{t}_{b}\\\:class-2,\:\:{t}_{a}\ge\:{t}_{b}\end{array}\right.$$

In SEFRON model, the performance can be optimized by choosing the suitable values of the tuning parameters, which are: number of receptive field neurons (Q), the overlap constant ($\:\beta\:$), the learning rate ($\:\mu\:$), the efficacy update range (σ), STDP learning window ($\:{\tau\:}_{+}$) and time constant of LIF neuron model ($\:\tau\:$). The effects of the parameters are analyzed in Sect. 3.

## Experimental results and discussion

As described in Sect. 2.1, two benchmark PD datasets from the UCI Machine Learning Repository were utilized to test and evaluate the performance of the proposed learning approach. The results obtained were then compared with existing learning methods designed for SNNs as well as traditional representative classifiers. The performance of the classifiers is evaluated using the accuracy, sensitivity, specificity, Matthew correlation coefficient (MCC), precision, F1 score and Gmean as figure of merits. As the first PD dataset used in this paper is imbalanced dataset, Gmean determines better robustness of the classifier than the accuracy. In this experiment, K-fold cross validation with different values of “K” (which are K = 3, 5, 8, and 10) is used and the average values of the performance measures are considered to evaluate the effectiveness of the classifiers. For the analysis, percentage splitting of the entire dataset into training and testing set (90% training set / 10% testing set, 85% training set / 15% testing set, 80% training set / 20% testing set, and 70% training set / 30% testing set) is also utilized. K-fold cross validation mitigates overfitting problem and provides better estimation on the reliability of the model than the single percentage split.

**Fig. 5:**
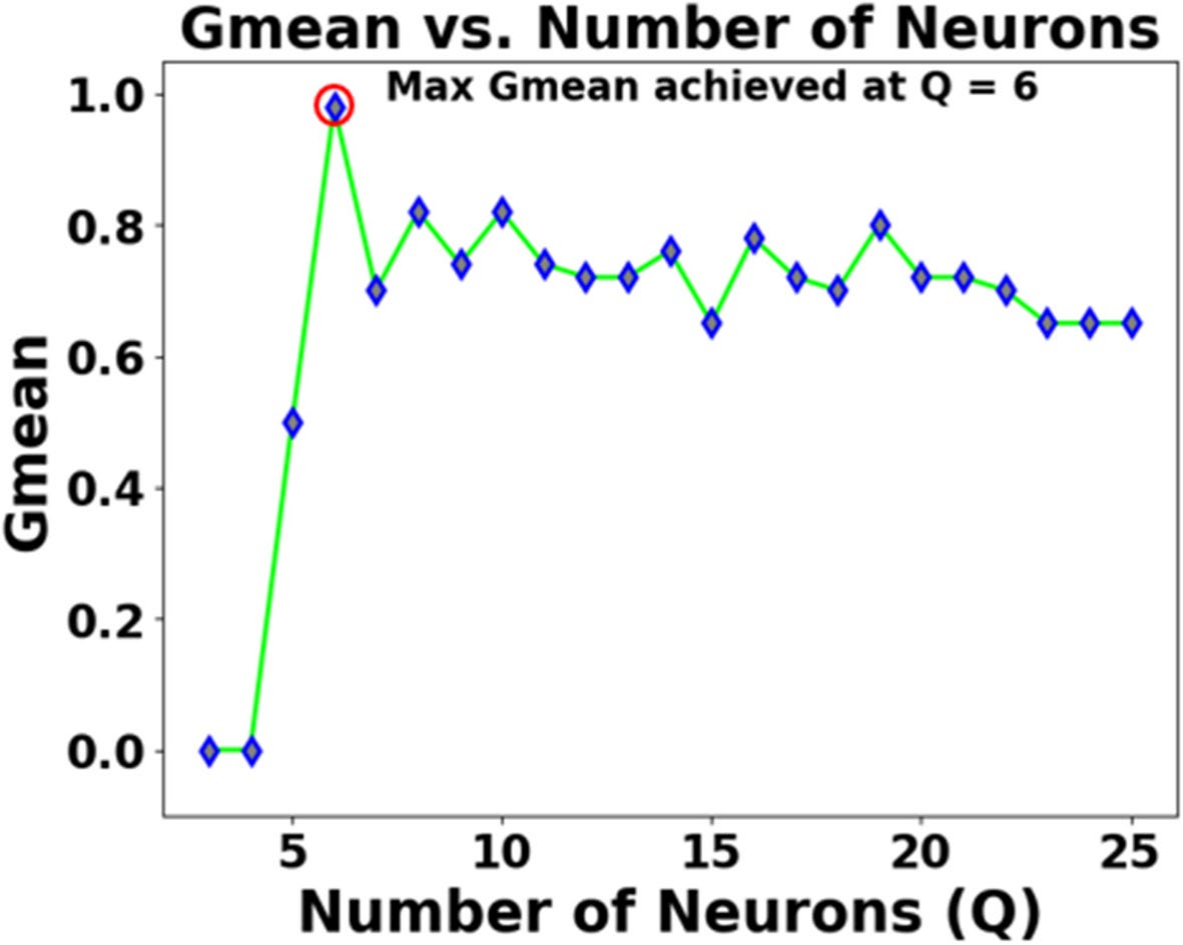
Impact of number of receptive field neurons ($\:Q$) on the Gmean [Dataset#1] of SEFRON

**Fig. 6:**
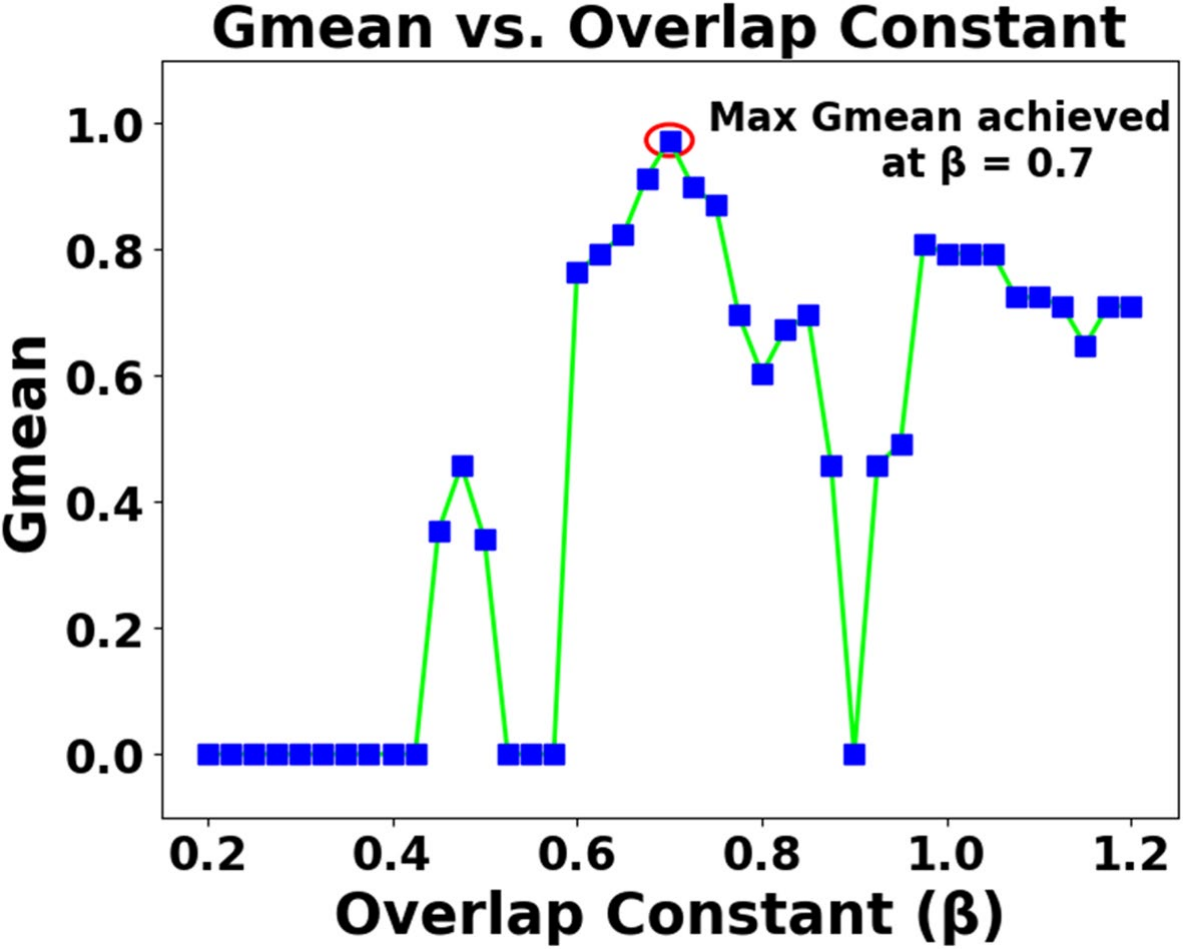
Impact of overlap constant ($\:\beta\:$) on the Gmean [Dataset#1] of SEFRON

**Fig. 7:**
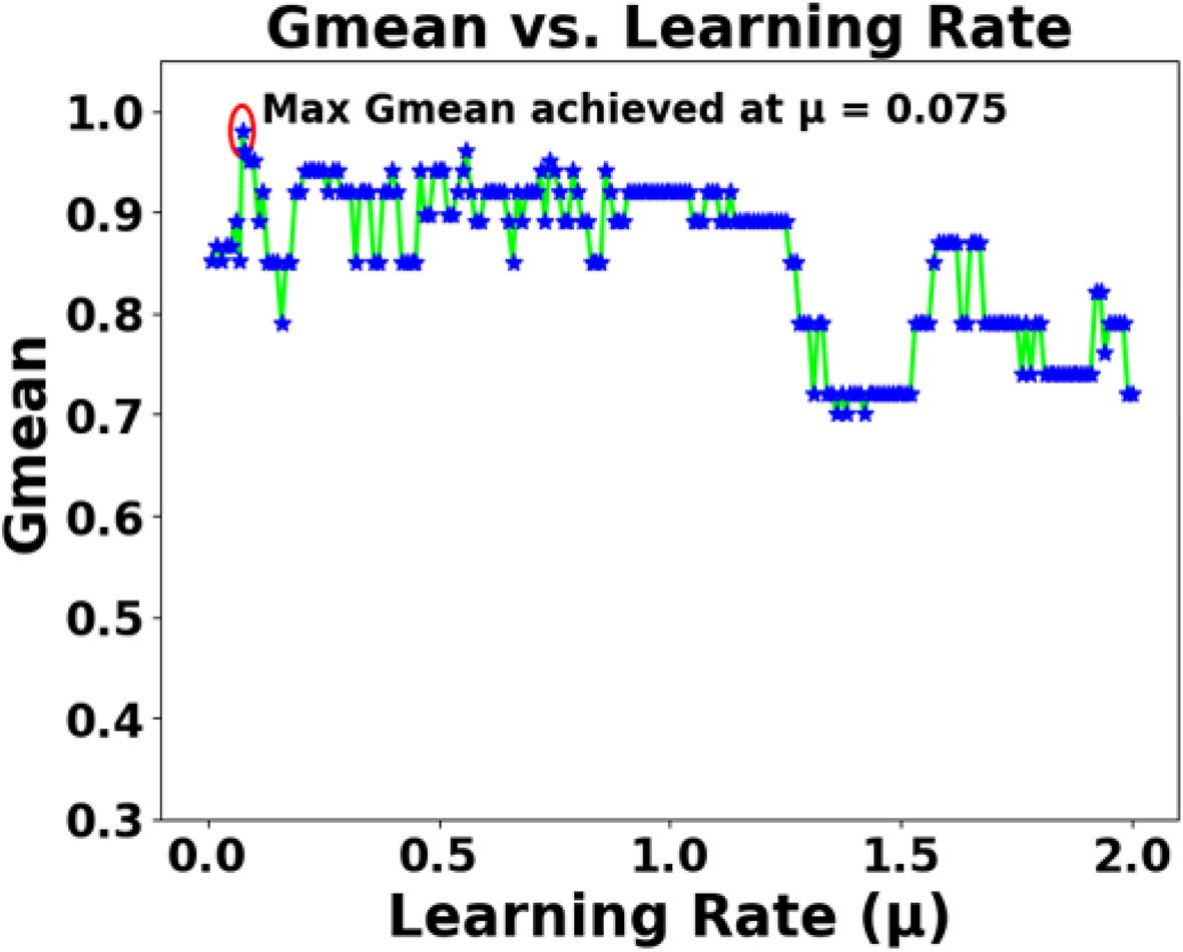
Impact of learning rate ($\:\mu\:$) on the Gmean [Dataset#1] of SEFRON

**Fig. 8:**
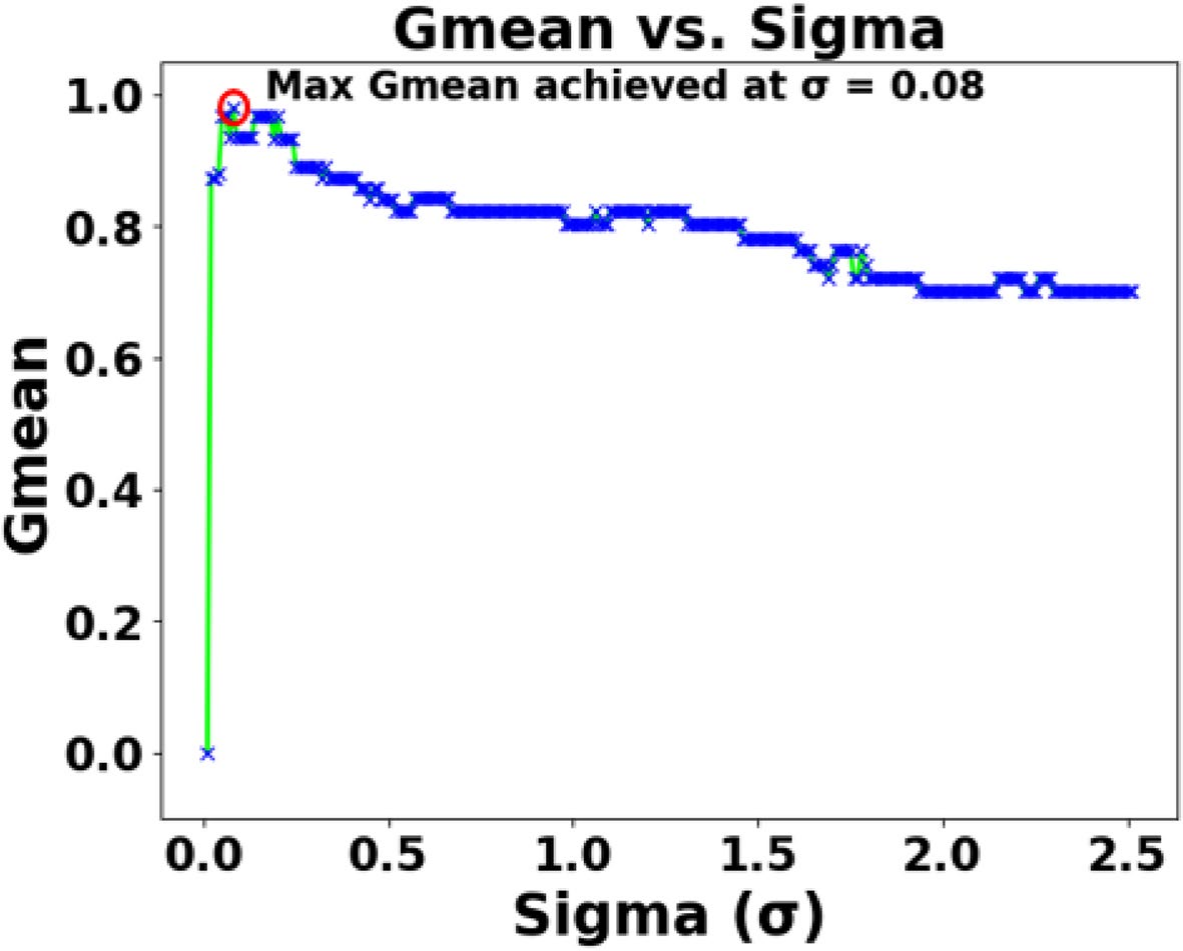
Impact of sigma (σ) on the Gmean [Dataset#1] of SEFRON

**Fig. 9:**
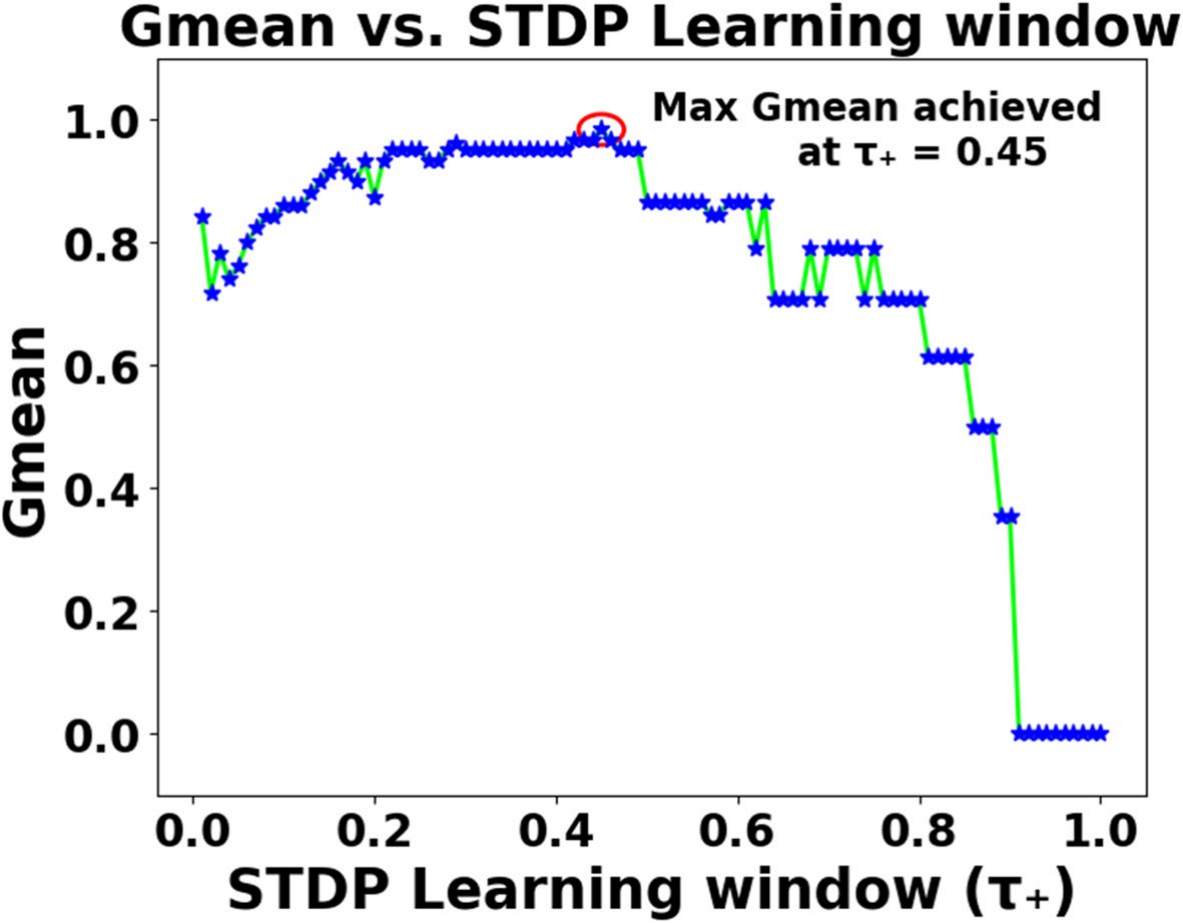
Impact of STDP learning window ($\:{\tau\:}_{+}$) on the Gmean [Dataset#1] of SEFRON

**Fig. 10:**
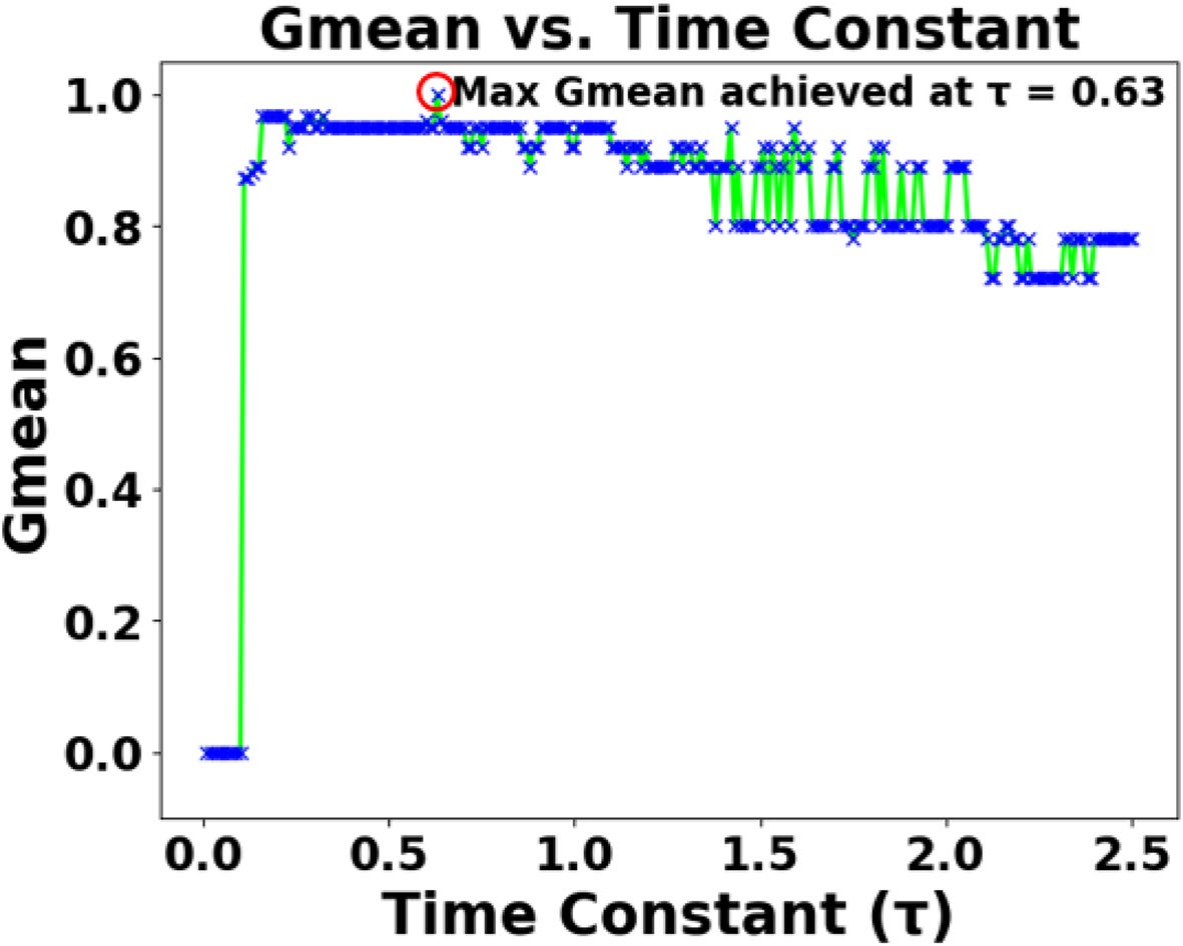
Impact of time constant ($\:\tau\:$) on the Gmean [Dataset#1] of SEFRON

As mentioned in Sect. 2.2, the effects of various parameters influencing the performance of SEFRON are studied thoroughly. The parameters are number of receptive field neurons ($\:Q$), the overlap constant ($\:\beta\:$), the learning rate ($\:\mu\:$), the efficacy update range (σ), STDP learning window ($\:{\tau\:}_{+}$) and time constant of LIF neuron model ($\:\tau\:$). The hyperparameter tuning is performed through a trial-and-error approach. A range of values is first chosen for each parameter, and their effects on the results are assessed. Then, different combinations of these parameter values are tested to achieve optimal performance. After getting the best result, the plots are done for a range of hyperparameter keeping other parameters to its optimum value. Hence, we can get the best visualization of the effect of each parameter on accuracy. For example: as per Table 3, the optimum values of the parameters are given as Q = 6, $\:\beta\:=0.7,\:\mu\:=0.075,\:{\upsigma\:}=0.08,\:\:{\tau\:}_{+}=0.45,\:\tau\:=0.63$. Now for the plot demonstrating the effect of the time constant, τ will vary from 0.1 to 2.5, while the other parameters remain fixed at Q = 6, $\:\beta\:=0.7,\:\mu\:=0.075,\:{\upsigma\:}=0.08,\:\:{\tau\:}_{+}=0.45$. Figure 5 shows how Gmean changes with the change in the number of receptive field neurons. The number of neurons indicates the data discriminability as well as the complexity of the model. As the number of neurons increases, the computational complexity also increases. Due to population encoding scheme, the minimum number of neurons ($\:Q$) is required to be 3 (see Eq. (3)). From Fig. 5, it is observed that the best performance is achieved for $\:Q=6$ and for $\:Q>6$ the performance slowly degrades. Figure 6 shows the effect of overlap constant on the performance of SEFRON. $\:\beta\:$ has an impact on the firing strength of receptive field neurons. It controls the width and hence, controls the localization of the spikes. Smaller width causes higher localization. In the study, $\:\beta\:$ varies from $\:0.2\:to\:1.2$ and the performance is evaluated for successive difference of 0.025. It has been seen that for $\:\beta\:<0.45$, Gmean is 0. This is because of the high localization due to small value of $\:\beta\:$ which causes underfitting. The optimum result is obtained for $\:\beta\:=0.7$. Learning rate plays an important role to determine the speed of the model. Higher the value of learning rate, faster the algorithm will converge but mean square error can also increase. Figure 7 shows that for $\:\mu\:>1.25$, the performance is degrading and for $\:\mu\:=0.075$, the Gmean is the highest. Although, this value is small indicating slow convergence, to design a highly precise classifier $\:\mu\:=0.075$ is considered for PD detection. Efficacy update range indicates the impact of weight change. If $\:\sigma\:\to\:{\infty\:}$, the synaptic efficacy function will no longer be time varying and it will become contact weight. Smaller the value of $\:\sigma\:$, more variation is present in the weight change. From Fig. 8, it can be seen that the best performance is obtained for $\:0.08\le\:\sigma\:\le\:0.16$; and for $\:\sigma\:\ge\:0.4$, Gmean degrades drastically. The effect of STDP learning window on the performance of SEFRON for the diagnosis of PD is depicted in Fig. 9. $\:{\tau\:}_{+}$ plays a crucial role in assessing the impact of each presynaptic spike on weight update. As $\:{\tau\:}_{+}$ increases, the contributions of presynaptic spikes occurring significantly earlier relative to the postsynaptic spike intensify, while those fired in closer proximity to the postsynaptic spike diminish. From Fig. 9, it can be seen that for $\:{0.35<\tau\:}_{+}<0.6$, favorable outcome is obtained. For $\:{\tau\:}_{+}>0.9$, Gmean is dropped to zero. Time constant of LIF neuron models the membrane potential decay time constant that determines the rise and decay time of the postsynaptic potential. Figure 10 shows the change in Gmean for $\:0.1\:ms\le\:\tau\:\le\:2.5\:ms$ and in this experiment, the value of the time constant is taken as $\:0.63\:ms$. Depending on the analysis, suitable values of the parameters are chosen and presented in Table 3.

**Table 3:** Parameter values chosen for SEFRON to detect Parkinson’s Disease

Parameters	Values	Parameters	Values
Q	6	σ	0.08
*β *	0.7	$\tau_+$	0.45
*µ*	0.075	$\tau$	0.63

**Fig. 11:**
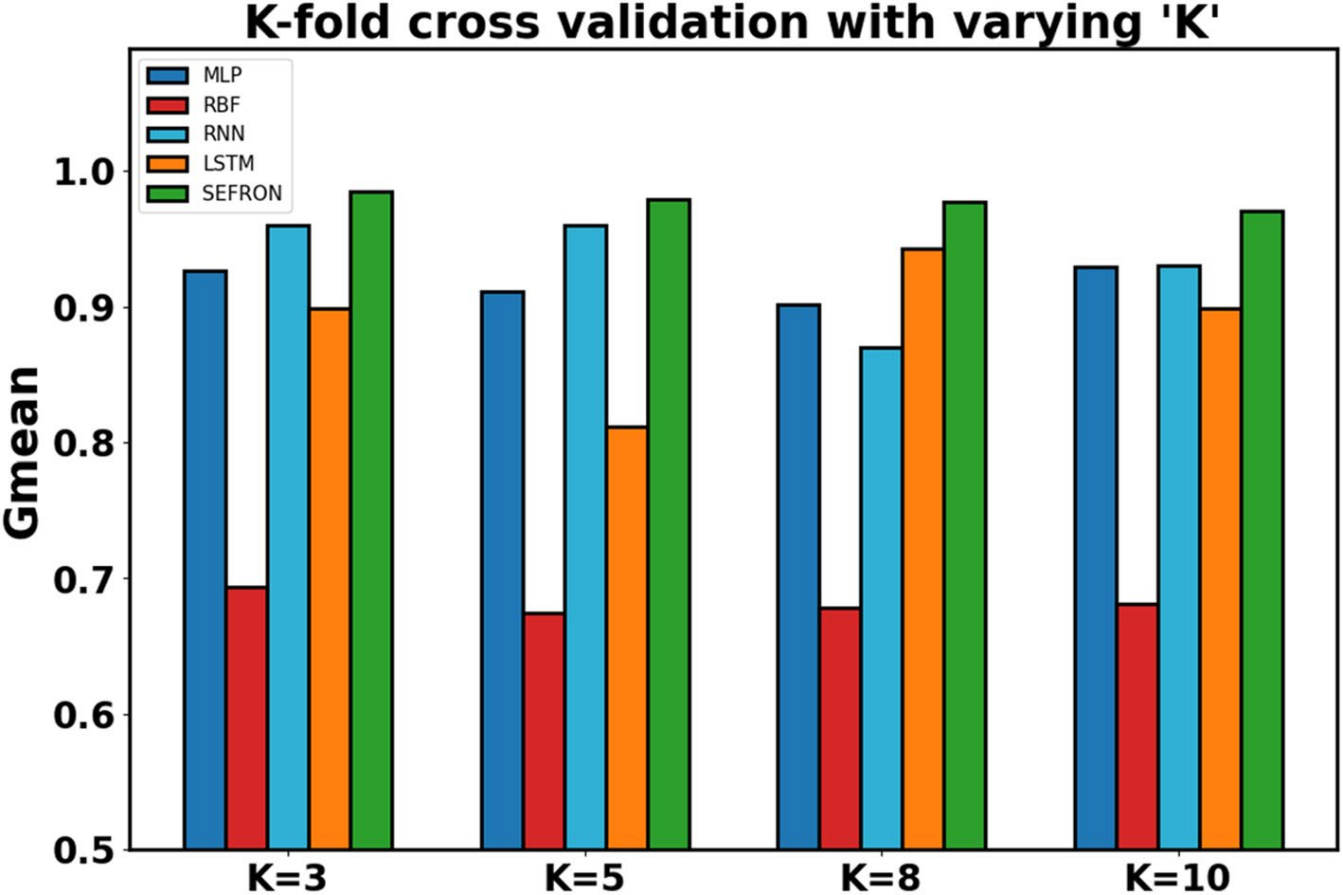
Performance comparison of MLP, RBF, RNN, LSTM and SEFRON using K-fold cross validation with different value of ‘K’ for dataset#1

**Fig. 12:**
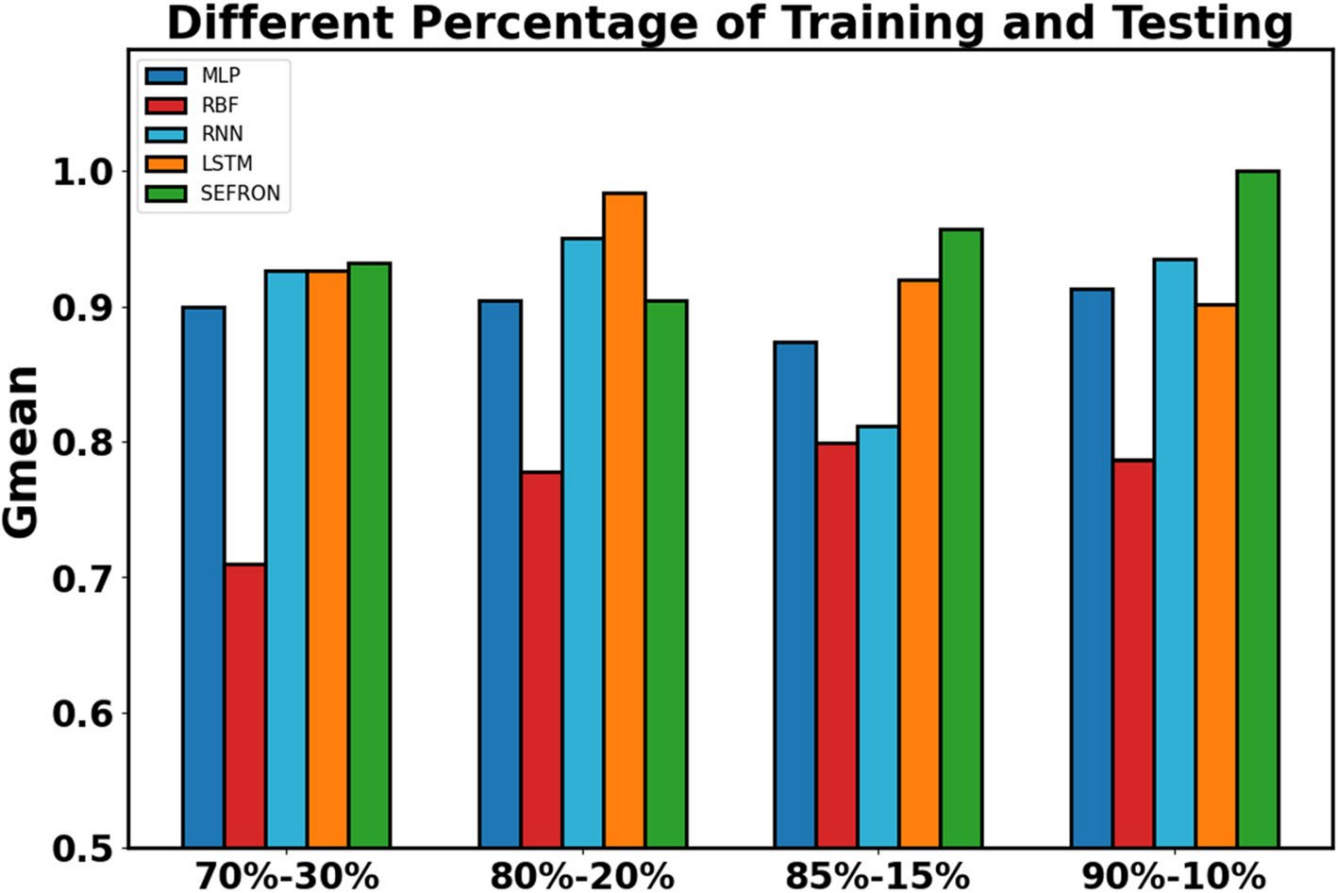
Performance comparison of MLP, RBF, RNN, LSTM and SEFRON with different percentage split of training and testing sets for dataset#1

**Fig. 13:**
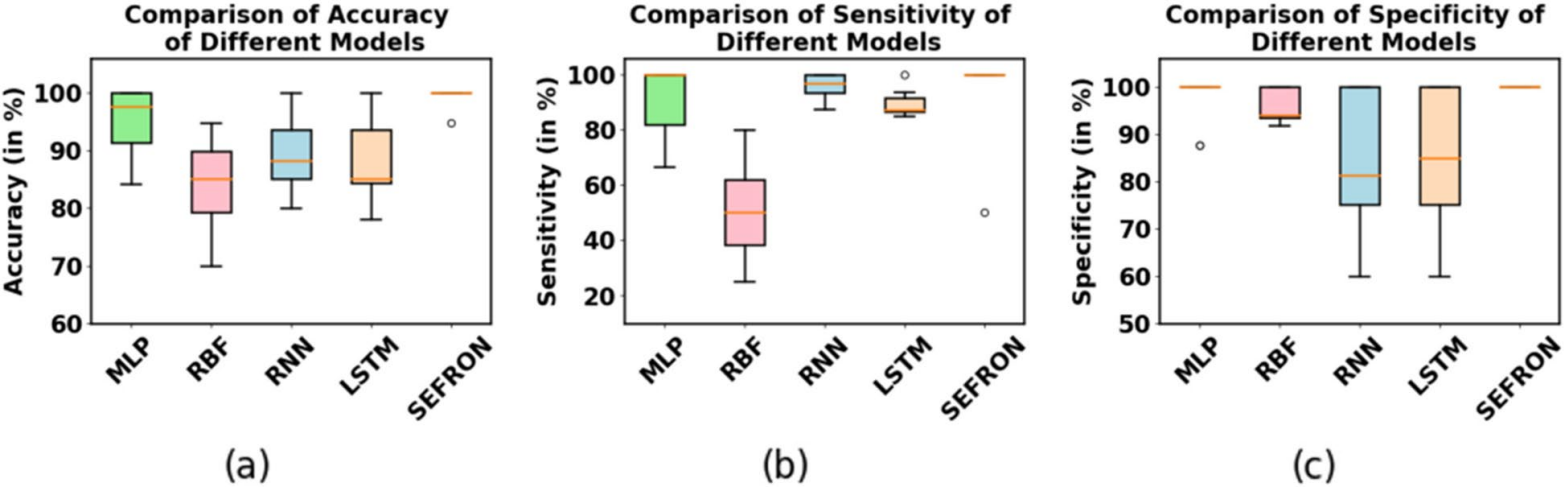
Comparison of box plots of (**a**) accuracy, (**b**) sensitivity and (**c**) specificity for MLP, RBF, RNN, LSTM and SEFRON using 10-fold cross validation for dataset #1

The presynaptic spike time interval ($\:T$) is chosen to be $\:3\:ms$. To capture the late postsynaptic spike, the time interval is chosen between $\:0\:to\:4\:ms$. PD detection being a 2-class problem, class-1 (PD patients) and class-2 (healthy subjects) are separated by a boundary ($\:{t}_{b}$) which is taken as $\:2\:ms$. The performance of SEFRON is compared with other neural network models: MLP-NN, RBF-NN, RNN and LSTM. In this experiment, the MLP-NN classifier has three hidden layers with 150, 100, and 50 neurons in first, second, and third layer respectively. The RBF-NN has 8 neurons in its single hidden layer. The RNN model has 64 neurons in SimpleRNN layer and 32 neurons in fully dense layer. In the LSTM model, each gate has 64 units that independently control the memory and output flow at each time step. Tables 4 and 5 present the performance of all mentioned models using K-fold cross validation and different percentage split respectively. In K-fold cross validation smaller the value of K, less number of samples are used to train the models. It can be seen that almost in every case, SEFRON outperforms other two models. The maximum average accuracy, sensitivity, specificity, and Gmean obtained by SEFRON is 99.49%, 96.97%, 100%, and 0.9845 for 3-fold cross validation. From Table 5, it can be seen that using 90% training set and 10% testing set, 100% accuracy is achieved by SEFRON model to separate PD patients from healthy subjects. Figure 11 shows the comparison of the accuracies for K-fold cross validation with varying K. The bar plot indicates that SEFRON outperforms other models for all values of K. Although, in Fig. 12, for 70% training and 30% testing set MLP-NN, RNN and LSTM have better accuracy than SEFRON, for other percentage splits, SEFRON proves to be the best classifier. Figure 13 present the comparison of the box plots of accuracy for MLP-NN, RBF-NN, RNN, LSTM and SEFRON for 10-fold cross validation. The width of the box shows the variation of the results. Larger width refers to high variation in the outcomes and narrow width indicates more reliable performance. In Fig. 13(a), it can be seen that the accuracy of RBF-NN varies the most and SEFRON gives consistent outcome. Similarly, Fig. 13(b) and Fig. 13(c) compares the boxplots of sensitivity and specificity respectively. Sensitivity of RBF-NN model is unsatisfactory (as seen in Fig. 13(b)). From Fig. 13(c), it is observed that MLP-NN and SEFRON performs well in terms of specificity but RNN and LSTM produces high range of specificity.

**Table 4:** Performance comparison for K-fold Cross Validation with different values of K for dataset #1

Technology	Accuracy (in %)	Sensitivity (in %)	Specificity (in %)	MCC	Precision	F1 Score	Gmean
K = 3							
MLP-NN	94.36	89.03	96.79	0.8502	0.8860	0.8798	0.9262
RBF-NN	84.61	51.32	95.06	0.5450	0.7906	0.6125	0.6937
RNN	96.5	94	98.05	0.95	0.9988	0.9682	0.96
LSTM	93.62	80.87	99.96	0.91	100	0.893	0.899
** SEFRON [Dataset#1]**	**99.49**	**96.97**	**100**	**0.9816**	**1**	**0.9841**	**0.9845**
K = 5							
MLP-NN	94.36	85.68	97.53	0.8373	0.8933	0.8641	0.9116
RBF-NN	83.08	49.62	93.61	0.4913	0.7110	0.5802	0.6743
RNN	96.67	95.83	98	0.9063	1.0	0.9787	0.969
LSTM	97.85	88.9	77	0.93	0.971	0.93	0.812
** SEFRON [Dataset#1]**	**99.48**	**96**	**100**	**0.9763**	**1**	**0.9778**	**0.9789**
K = 8							
MLP-NN	94.35	83.54	98.09	0.8432	0.9292	0.8700	0.9019
RBF-NN	83.63	49.27	94.15	0.5208	0.7833	0.5978	0.6780
RNN	91.94	80.4	98.21	0.82	0.77	0.89	0.87
LSTM	95.52	92.84	98	0.9017	0.9622	0.93	0.943
** SEFRON [Dataset#1]**	**98.96**	**95.63**	**100**	**0.9702**	**1**	**0.9756**	**0.9768**
K = 10							
MLP-NN	95.39	88.42	98.13	0.8756	0.93	0.8997	0.9294
RBF-NN	84.68	49.67	95.95	0.5270	0.7583	0.5946	0.6809
RNN	92.11	100	88.69	0.84	0.78	0.86	0.93
LSTM	93.87	84.56	98.33	0.90	0.9273	0.89	0.899
** SEFRON [Dataset#1]**	**99.47**	**95**	**100**	**0.9687**	**1**	**0.9667**	**0.9708**

**Table 5:** Performance comparison with different percentage split for dataset #1

Technology	Accuracy (in %)	Sensitivity (in %)	Specificity (in %)	MCC	Precision	F1 Score	Gmean
90% training set − 10% testing set							
MLP-NN	95	83.33	100	0.8819	1	0.9091	0.9129
RBF-NN	85	66.67	92.86	0.6299	0.8	0.7273	0.7868
RNN	90	87.5	100	0.7638	1.0	0.9333	0.9354
LSTM	85	81.25	100	0.6813	1.0	0.8965	0.9014
** SEFRON [Dataset#1]**	100	100	100	1	1	1	1
85% training set − 15% testing set							
MLP-NN	90	83.33	91.67	0.7092	0.7143	0.7692	0.8740
RBF-NN	90	66.67	95.83	0.6708	0.8	0.7273	0.7993
RNN	88.7	66	100	0.7512	1.0	0.7911	0.812
LSTM	89.72	100	84.3	0.808	0.7767	0.8743	0.92
** SEFRON [Dataset#1]**	**93.33**	**100**	**91.67**	**0.8292**	**0.75**	**0.8571**	**0.9574**
80% training set − 20% testing set							
MLP-NN	92.31	87.5	93.55	0.7767	0.7778	0.8235	0.9047
RBF-NN	89.74	62.5	96.77	0.6633	0.8333	0.7143	0.7777
RNN	92.86	100	90.11	0.85	0.80	0.89	0.95
LSTM	90	74.5	94.24	0.9274	1.0	0.9836	0.9837
** SEFRON [Dataset#1]**	**92.31**	**87.5**	**93.55**	**0.7767**	**0.7778**	**0.8235**	**0.9047**
70% training set − 30% testing set							
MLP-NN	93.22	84.62	95.65	0.8027	0.8462	0.8462	0.8996
RBF-NN	84.74	53.85	93.47	0.5223	0.7	0.6087	0.71
RNN	93.22	93.61	91.67	0.8068	0.9778	0.9565	0.9263
LSTM	93.22	93.61	91.67	0.8068	0.9778	0.9565	0.9263
** SEFRON [Dataset#1]**	**89.83**	**100**	**86.96**	**0.7713**	**0.6842**	**0.8125**	**0.9325**

The performance comparison of various technologies using K-fold cross-validation and different percentage splits for dataset 2 reveals several noteworthy observations. In Table 6, the SEFRON model consistently outperformed others across all values of K, achieving the highest accuracy, sensitivity, specificity, and F1 score, particularly at K = 10 with an accuracy of 91.94%. This indicates its robustness in classifying the data effectively. Meanwhile, the RBF-NN also showed commendable results, especially with a K of 5, where it recorded an accuracy of 84.11% and high sensitivity. Table 7 highlights the performance of the classifiers based on different training and testing percentages. Notably, the SEFRON model again excelled with 89.58% accuracy when using an 80%−20% split, indicating its reliability in scenarios with varying training data proportions. The RBF-NN demonstrated strong performance as well, particularly at an 85%−15% split, where it reached an accuracy of 83.33%. Overall, both tables suggest that while SEFRON emerges as the superior model, RBF-NN and RNN also deliver competitive results. Table 8 compares the suggested model with some state-of-the-art SNN models used for the detection of PD. Despite variations in the datasets, an attempt has been made to offer insight into the current developments within this field. It can be seen that the proposed model demonstrates superior performance compared to other SNN models as well.

In summary, SEFRON classifier achieves better performance compared to other classifiers. Also, the computational complexity is less in SEFRON. 100% accuracy is obtained by SEFRON by using only 6 receptive field neurons whereas MLP-NN has 150, 100, 50 neurons in three hidden layers. Although RBF-NN has only 8 neurons in the hidden layer, performance is poor in comparison to SEFRON.

**Table 6:** Performance comparison for K-fold cross validation with different values of K for dataset #2

Technology	Accuracy (in %)	Sensitivity (in %)	Specificity (in %)	MCC	Precision	F1 Score	Gmean
K = 3							
MLP-NN	78.45	81.23	74.65	0.5612	0.8023	0.8345	0.7864
RBF-NN	81.27	76.54	85.12	0.6138	0.8432	0.8117	0.798
RNN	80.02	80.55	79.42	0.5914	0.8056	0.8015	0.8002
LSTM	79.03	73.12	84.78	0.5784	0.7986	0.7623	0.7664
** SEFRON [Dataset#2]**	**86.12**	**85.23**	**87.34**	**0.7325**	**0.8724**	**0.8506**	**0.8652**
K = 5							
MLP-NN	77.88	75.34	78.56	0.5431	0.791	0.7709	0.7736
RBF-NN	84.11	90.67	77.25	0.6659	0.8154	0.8523	0.8459
RNN	84.03	82.47	86.65	0.7199	0.8412	0.835	0.8325
LSTM	81.67	78.22	82.4	0.6197	0.8061	0.8305	0.8183
** SEFRON [Dataset#2]**	**88.03**	**86.12**	**89.85**	**0.7551**	**0.9023**	**0.8825**	**0.8861**
K = 8							
MLP-NN	82.14	78.67	85.42	0.6115	0.8189	0.8027	0.8049
RBF-NN	86.32	81.25	92.1	0.7424	0.9057	0.8615	0.8722
RNN	84.03	82.47	86.65	0.7199	0.8412	0.835	0.8325
LSTM	83.09	80.21	85.18	0.6743	0.795	0.8202	0.8111
** SEFRON [Dataset#2]**	**90.14**	**89.45**	**91.32**	**0.7921**	**0.915**	**0.8913**	**0.8957**
K = 10							
MLP-NN	83.33	79.32	88.67	0.6478	0.85	0.81	0.816
RBF-NN	85.22	84.45	90.13	0.7115	0.9	0.87	0.877
RNN	86	88.77	86.5	0.7521	0.873	0.8714	0.8750
LSTM	84.53	86.67	82.22	0.6814	0.7905	0.8327	0.8141
** SEFRON [Dataset#2]**	**91.94**	**99.95**	**87.69**	**0.82**	**0.77**	**0.89**	**0.87**

**Table 7:** Performance comparison with different percentage Split for dataset #2

Technology	Accuracy (in %)	Sensitivity (in %)	Specificity (in %)	MCC	Precision	F1 Score	Gmean
90% training set − 10% testing set							
MLP-NN	79.16	84.61	72.73	0.5795	0.7857	0.8148	0.7844
RBF-NN	80.56	77.78	83.33	0.612	0.8223	0.7999	0.8050
RNN	79.17	72.72	84.62	0.5795	0.8	0.7619	0.7844
LSTM	79.17	81.82	76.92	0.5853	0.75	0.7826	0.7933
** SEFRON [Dataset#2]**	**85.41**	**82.61**	**88**	**0.7079**	**0.8636**	**0.8445**	**0.8526**
85% training set − 15% testing set							
MLP-NN	77.78	73.68	82.35	0.5604	0.8235	0.7778	0.7789
RBF-NN	83.33	92.30	72.72	0.6693	0.8	0.8571	0.7844
RNN	72.22	66.67	77.78	0.4472	0.75	0.7059	0.72
LSTM	80.56	77.78	83.33	0.612	0.823	0.799	0.805
** SEFRON [Dataset#2]**	**86.61**	**84.21**	**88.23**	**0.7233**	**0.1389**	**0.8649**	**0.8619**
80% training set − 20% testing set							
MLP-NN	81.25	78.26	84	0.6242	0.8181	0.8	0.8108
RBF-NN	87.5	82.61	92	0.7513	0.9048	0.8260	0.8636
RNN	81.25	83.33	79.17	0.6255	0.8	0.8163	0.8122
LSTM	81.25	87.5	75	0.6299	0.7778	0.8235	0.81
** SEFRON [Dataset#2]**	**89.58**	**91.3**	**88**	**0.7923**	**0.875**	**0.8936**	**0.8963**
70% training set − 30% testing set							
MLP-NN	83.33	77.78	88.89	0.6708	0.875	0.823	0.8314
RBF-NN	84.72	77.78	91.68	0.7012	0.9032	0.8358	0.8443
RNN	87.5	89.19	85.71	0.7499	0.8684	0.88	0.8743
LSTM	83.33	89.19	77.14	0.6696	0.8049	0.8461	0.8295
** SEFRON [Dataset#2]**	**88.89**	**89.47**	**88.23**	**0.7771**	**0.8947**	**0.8947**	**0.8885**

**Table 8:** Performance comparison with other state-of-the-art SNN models

Reference and Year	Dataset Used	Model/Algorithm Used	Accuracy (in %)
López-Vázquez et al. [36], 2019	UCI Machine Learning Repository for PD	Grammatical Evolution (GE)-based SNN	88.75%
Kerman et al. [37], 2022	Spike data collected from different regions of Brain	Spiking MLP	93%
Siddique et al. [38], 2023	Spike data from the neurons in the subthalamic nucleus region	Spiking LSTM	99.48%
**Proposed model [Dataset#1]**	**UCI Machine Learning Repository for PD **[51]	**Time-varying Synaptic Efficacy Function based SNN (SEFRON)**	**100%**
**Proposed model [Dataset#2]**	**UCI Machine Learning Repository: Parkinson Dataset with replicated acoustic features **[52]	**Time-varying Synaptic Efficacy Function based SNN (SEFRON)**	**91.94%**

## Conclusion

An automatic PD detection technique which is compatible with neuromorphic devices is in dire need to control the mentioned disease. Therefore, in this study, the SEFRON model is investigated and compared with other neural network architectures (MLP, RBF, RNN, and LSTM). The experimental results revealed that SEFRON outperforms other neural networks with maximum accuracy of 100% and average accuracy of 99.49% using k-fold cross validation for dataset 1 and maximum accuracy of 94% and an average accuracy of 91.94% for dataset 2, thus making it acceptable for clinical trial. One significant limitation of our study is the relatively small sample size of our dataset. It has impact on the performance metrics such as precision. Notably, there are variations in precision as the proportion of the testing set increased, underscoring the sensitivity of our model to dataset distribution. Conducting independent analysis of datasets 1 and 2 may increase the risk of SEFRON overfitting, as it prevents validation across diverse data sources and could limit generalizability. However, the datasets are analyzed separately because they differ in terms of data collection methods, population size, and feature structure. Dataset 1 includes 195 instances with 22 features from 31 individuals, while dataset 2 has 240 instances with 45 features from 80 individuals with three replicated recordings per subject. These differences could impact model performance if combined. Although, cross-dataset validation could help address generalizability and overfitting problems, due to the differences in inherent structure, combining the datasets could introduce biases. Hence, cross-dataset validation will be studied in future work using more harmonized datasets to strengthen SEFRON’s robustness. Additionally, different SNN based models will also be studied to further reduce the structural complexity and improve the computational power.

## Data Availability

“The dataset generated and/or analyzed during the current study are available in the UCI: Oxford Parkinson’s Disease Detection repository 10.24432/C59C74 [42].”
